# Post cardiac surgery vasoplegia is associated with high preoperative copeptin plasma concentration

**DOI:** 10.1186/cc10516

**Published:** 2011-10-25

**Authors:** Pascal H Colson, Cedric Bernard, Joachim Struck, Nils G Morgenthaler, Bernard Albat, Gilles Guillon

**Affiliations:** 1 Department of Anesthesia and Intensive Care, Hôpital Arnaud de Villeneuve, F-34295 Montpellier, France; 2 Institut de Génomique Fonctionnelle, Département d'Endocrinologie, CNRS UMR 5203, INSERM U 661, Université Montpellier I and Université Montpellier II, F-34094 Montpellier, France; 3 Department of Research, Thermo FisherScientific, Neuendorfstr. 25, 16761 Hennigsdorf bei Berlin, Germany; 4 Department of Cardiothoracic and Vascular Surgery, Hôpital Arnaud de Villeneuve, F-34295 Montpellier, France

## Abstract

**Introduction:**

Post cardiac surgery vasodilatation (PCSV) is possibly related to a vasopressin deficiency that could relate to chronic stimulation of adeno-hypophysis. To assess vasopressin system activation, a perioperative course of copeptin and vasopressin plasma concentrations were studied in consecutive patients operated on for cardiac surgery.

**Methods:**

Sixty-four consecutive patients scheduled for elective cardiac surgery with cardiopulmonary bypass were studied. Hemodynamic, laboratory and clinical data were recorded before and during cardiopulmonary bypass, and at the eighth postoperative hour (H8). At the same time, blood was withdrawn to determine plasma concentrations of arginine vasopressin (AVP, radioimmunoassay) and copeptin (immunoluminometric assay). PCSV was defined as mean arterial blood pressure < 60 mmHg with cardiac index ≥ 2.2 l/min/m^2^, and was treated with norepinephrine to restore mean blood pressure > 60 mmHg. Patients with PCSV were compared with the other patients (controls). Student's *t *test, Fisher's exact test, or nonparametric tests (Mann-Whitney, Wilcoxon) were used when appropriate. Correlation between AVP and copeptin was evaluated and receiver-operator characteristic analysis assessed the utility of preoperative copeptin to distinguish between controls and PCSV patients.

**Results:**

Patients who experienced PCSV had significantly higher copeptin plasma concentration before cardiopulmonary bypass (*P *< 0.001) but lower AVP concentrations at H8 (*P *< 0.01) than controls. PCSV patients had preoperative hyponatremia and decreased left ventricle ejection fraction, and experienced more complex surgery (redo). The area under the receiver-operator characteristic curve of preoperative copeptin concentration was 0.86 ± 0.04 (95% confidence interval = 0.78 to 0.94; *P *< 0.001). The best predictive value for preoperative copeptin plasma concentration was 9.43 pmol/l with a sensitivity of 90% and a specificity of 77%.

**Conclusions:**

High preoperative copeptin plasma concentration is predictive of PSCV and suggests an activation of the AVP system before surgery that may facilitate depletion of endogenous AVP stores and a relative AVP deficit after surgery.

## Introduction

Severe postoperative cardiovascular failure occurs in 3 to 5% of patients undergoing cardiac surgery using cardiopulmonary bypass (CPB) [1,2]. In some cases, a post cardiotomy vasodilatory shock characterized by a decreased vascular tone has been reported that might be explained by inadequate plasma concentrations of the vasoconstrictor hormone arginine vasopressin (AVP) [3-5]. Accordingly, first clinical reports showed beneficial effects of AVP treatment in cardiac surgery patients with severe vasodilatory shock [6,7] that correlates with results observed in septic shock where a deficiency in vasopressin has been described [8,9]. However, why some patients are prone to develop a deficiency in vasopressin after cardiac surgery, and whether postoperative vasodilatation without shock (vasoplegia) is associated with vasopressin deficiency, remain unclear.

Generation of copeptin correlates with the release of vasopressin, since both are derived from the same precursor. Furthermore, the stability of the precursor protein was found to be high and fully suitable for routine purposes. Copeptin is therefore easier to measure than vasopressin, and could be used as a marker of vasopressin release [5,10]. High copeptin concentration has been observed in chronic heart failure and acute myocardial infarction [11,12]. There are robust data showing that vasopressin is related to heart failure severity and outcome [13]. A prognosis-predictive value of copeptin has also been shown in critically ill patients [10,11,14,15], in coronary artery disease [16] and in advanced heart failure [17].

We hypothesized that patients who develop post cardiac surgery vasodilation (PCSV) may have an activation of the vasopressin system before surgery that favors a relative deficit in vasopressin after surgery. We have analyzed the kinetics of plasma concentrations of copeptin and AVP before, during and just after cardiac surgery in patients undergoing elective uncomplicated cardiac surgery, and compared patients with PCVS syndrome with the other patients.

## Materials and methods

### Patient selection and management

The observational study was designed to collect perioperative data from consecutive patients operated on for elective cardiac surgery with CPB until a total of 10 patients experiencing PCSV (see definition below) had been included. Patients suffering from chronic renal failure and under dialysis, and patients who underwent complicated surgical course (significant bleeding requiring reoperation, low cardiac output requiring high inotrope dose exceeding dobutamine > 10 μg/kg/minute or any dose of adrenaline and/or mechanical circulatory assistance) were not included. Comprehensive preoperative data were collected from cardiac and medical histories for all patients. All patients gave written informed consent for collecting their perioperative records, and blood samples for AVP and copeptin measurements were obtained from plasma processed for routine biological measurements. The protocol met the criteria of a noninterventional study design as defined by French law and has been approved by the Institutional Review Board of Centre Hospitalier Universitaire de Nîmes.

The usual cardiac treatment was maintained until the day before surgery. Perioperative management (anesthesia, CPB, and cardioplegia) is standardized for all patients. General anesthesia was induced and maintained with titrated propofol and sufentanil. Cisatracurium was used to facilitate endotracheal intubation. After tracheal intubation, ventilation was controlled to ensure normal blood gases using an inspired oxygen concentration of 50% (oxygen-air mixture) before CPB, and 100% oxygen after separation from bypass. In all patients, a peripheral vein was cannulated before anesthesia, and an arterial radial catheter was inserted after induction of anesthesia for continuous monitoring of mean arterial pressure (MAP). Before CPB, hypertension and hypotension were defined as an increase or a decrease in MAP of ≥ 20% from baseline (preinduction MAP), respectively. Hypertension was treated with additional doses of sufentanil. Hypotension was treated with rapid intravenous administration of lactated Ringer's solution. Ephedrine (3 mg bolus) could be used when MAP was ≤ 60 mmHg. After aortic and right atrium cannulation, CPB was instituted with a membrane oxygenator primed with 1.5 l crystalloid, and body temperature was maintained at 34 to 36°C. After aortic cross-clamping, a hyperkalemic blood cardioplegic solution was infused into the aortic root of the aorta until myocardial arrest. A nonpulsatile pump flow rate was maintained between 2.2 and 2.6 l/min/m^2^, provided the mixed venous oxygen saturation monitored on the pump machine exceeded 60%. After completion of the surgical procedure, patients were weaned from CPB when a rectal temperature of 36°C had been reached.

In the ICU, inotropic support (dobutamine) was used in order to obtain a cardiac index output ≥ 2.2 l/min/m^2 ^as assessed by thermodilution (pulmonary catheter) or echocardiography (aortic flow measured by pulsed Doppler time velocity integral). Hypotension (MAP ≤ 60 mmHg) without cardiogenic shock features (all patients had cardiac index > 2.2 l/min/m^2^) defined PCSV, and was treated with continuous intravenous administration of norepinephrine in order to restore MAP > 60 mmHg. Weaning from the ventilator was started during emergence of anesthesia, and extubation occurred when stable hemodynamics and normothermia had been maintained for ≥ 1 hour under propofol infusion; repeated boluses of morphine were used to keep patients pain-free.

### Data collection

For all patients, demographic data, past medical history, chronic intake of cardiac drugs or drugs with possible interference with sodium balance (diuretics), preoperative ejection fraction, type of surgical intervention, and the aortic cross-clamp and surgery times were documented.

The MAP and heart rate were monitored continuously. Laboratory parameters (hemoglobin, serum osmolarity, creatinine, electrolytes, arterial lactate, and arterial blood gas analysis), urine output, administration of ephedrine, diuretics, dobutamine or norepinephrine, and the need for volume loading were recorded during surgery and during the first 8 postoperative hours.

Blood samples were obtained from routine blood withdrawals before CPB (T0), during CPB and after surgery, at the eighth postoperative hour (H8), without extra blood subtraction. These samples were used for AVP and copeptin measurements.

### Measurement of AVP and copeptin plasma concentrations

Blinded frozen plasma samples were transferred to the endocrinology laboratories. For measurement of AVP, 1 ml ethylenediamine tetraacetic acid plasma was extracted with 4 ml ethanol, evaporated, and then reconstituted in 1 ml assay buffer and 0.3 ml extract. Subsequently, 0.4 ml extract was assayed using a radioimmunoassay (Bühlman Laboratories AG, Schonenbuch, Switzerland). The AVP assay standard calibration curve ranges from 1.25 to 80 pg/ml with a minimum quantization limit of 0.75 pg/ml. The intra-assay and inter-assay variation is 6.0% and 9.9%, respectively. Only when test results lay outside the clinically expected range (< 1.25 pg/ml or > 80 pg/ml) were measurements repeated to confirm the results. All other measurements were performed once.

Copeptin plasma concentrations were determined using a sandwich immunoluminometric assay (B.R.A.H.M.S. Copeptin LIA; Thermo Fisher Scientific, Hennigsdorf/Berlin, Germany). The lower detection limit was 0.4 pmol/l and the functional assay sensitivity (20% inter-assay coefficient of variation) was < 1 pmol/l.

### Statistical analysis

The 10 patients with PCSV were compared with the other patients (control group). For statistical analysis, the SPSS software program (Version SigmatStat 3.0; SPSS Inc., San Jose, CAL, USA) and XLSTAT (version 2011.3.01; Addinsoft, Paris, France) were used. Normality distribution was assessed by a Kolmonorov-Smirnov test. Most variables showed deviation from normality and were analyzed with nonparametric tests (Mann-Whitney or Wilcoxon tests). Few variables showed a normal distribution and were analyzed with Student's *t *tests, but all data are expressed as the median (25th to 75th percentiles) to simplify presentation. A Fisher's exact test was performed to analyze proportions and rates. For multiple comparisons (*n *= 3 for AVP or copeptin measurements), a Friedman repeated-measure analysis of variance with a Dunn method was used within groups and a Bonferroni correction was used for comparison between groups (statistical significance was then assumed if *P *≤ 0.016). A Spearman rank-order correlation test was performed to assess the correlation between AVP and copeptin plasma concentrations in all study patients.

Receiver-operator characteristic analysis (area under the curve, with 95% confidence interval) was calculated to assess the utility of preoperative copeptin to distinguish between controls and PCSV patients. We considered *P *≤ 0.05 as statistically significant.

## Results

**Figure 1:**
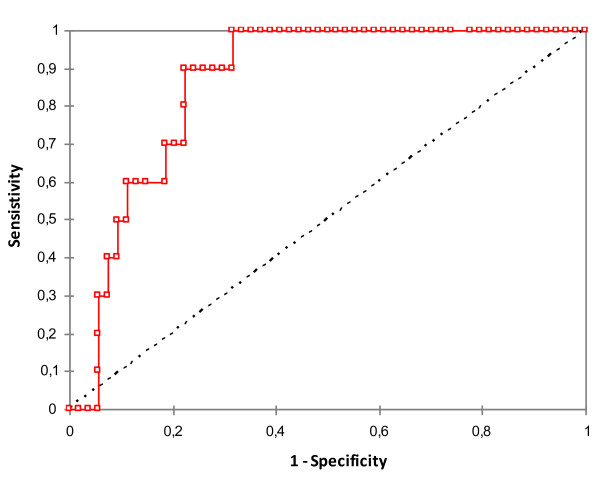
**Receiver-operator characteristic analysis of preoperative copeptin**. Receiver-operator characteristic analysis (area under the curve with 95% confidence interval) to assess the utility of preoperative copeptin to distinguish between controls and post cardiac surgery vasodilatation patients. Receiver-operator characteristic area under the curve was 0.86 ± 0.04 (95% confidence interval = 0.78 to 0.94; *P *< 0.001).

Sixty-four patients were included within 2 months, of which 10 were treated with norepinephrine in the ICU. Preoperative data for the two groups are summarized in Table 1. Patients who experienced PCSV were more often in New York Heart Association functional class IV (50% vs. 7.4%, respectively; *P *= 0.01), had lower preoperative left ventricle ejection fraction and natremia, were more frequently treated with diuretics, and had a higher copeptin levels before CPB than controls. The area under the receiver-operator characteristic curve for preoperative copeptin concentration was 0.86 ± 0.04 (95% confidence interval = 0.78 to 0.94; *P *< 0.001) (Figure 1). The best predictive value for preoperative copeptin plasma concentration was 9.43 pmol/l with a sensitivity of 90% and a specificity of 77% (positive predictive value = 43%, negative predictive value = 98%).

**Table 1:** Preoperative characteristics

	PCSV patients (*n *= 10)	Controls (*n *= 54)	*P *value
Age (years)	70 (57 to 77)	67 (60 to 76)	0.71
Male (%)	7 (70)	36 (67)	1.00
Body mass index	25.7 (23.1 to 28.6)	26.3 (24.3 to 28.0)	0.62
Preoperative status			
LVEF (%)	50 (30 to 60)	60 (50 to 70)	0.03
Urea (mmol/l)	7.3 (6.4 to 15.2)	7.3 (5.9 to 10.8)	0.38
Na^+ ^(mmol/l)	137 (134 to 139)	140 (138 to 141)	0.01
Osmolarity (mOsmol/l)	292 (284 to 296)	294 (290 to 298)	0.06
Hemoglobin (g/dl)	12.3 (10.1 to 15.0)	13.2 (11.7 to 14.6)	0.32
Platelets (10^9^/l)	196.5 (131 to 241)	219.5 (181 to 252)	0.10
Comorbidities			
Hypertension (%)	5 (50)	24 (44)	0.75
Diabetes (%)	4 (40)	7 (13)	0.06
Euroscore	9.5 (6 to 14)	6 (5 to 8)	0.02
Preoperative treatments			
Beta-blocker (%)	4 (40)	28 (52)	0.73
ACE inhibitor or sartan (%)	5 (50)	37 (69)	0.29
Calcium antagonist (%)	1 (10)	11 (20)	0.67
Diuretic (%)	9 (90)	26 (48)	0.02
Statin (%)	6 (60)	32 (59)	1.00
AVP and copeptin			
AVP (pmol/ml)	4.3 (1.4 to 6.3)	2.1 (0.9 to 5.1)	0.34
Copeptin (pmol/l)	30.1 (9.8 to 69)	4.8 (3.0 to 9.2)	< 0.001

Data expressed as median (25th to 75th percentiles) or number (%). ACE, angiotensin-converting enzyme inhibitor; AVP, arginine vasopressin; LVEF, left ventricle ejection fraction; PCSV, post cardiac surgery vasodilatation.

Surgery procedure details are reported in Table 2. PCSV patients had longer CPB (191 minutes (150 to 280) vs. 103 minutes (87 to 159), *P *< 0.001) and clamping times (75.5 minutes (58 to 104) vs. 138.5 minutes (115 to 192), *P *< 0.001) than controls, but no significant hemodynamic differences during surgery and CPB. However, they more often needed inotropic support with moderate doses of dobutamine to achieve adequate cardiac output after CPB weaning (8/10 vs. 4/54, *P *< 0.001) (Table 2). The AVP plasma concentration increased significantly between T0 and CPB in controls (*P *< 0.016) but not in PCSV patients. Copeptin plasma levels did not increase significantly in either group between T0 and CPB but were significantly higher in the PCSV patients than in controls.

**Table 2:** Intraoperative data

	PCSV patients (*n *= 10)	Controls (*n *= 54)	*P *value
Surgery			
Ascending aorta (%)	2 (20)	7 (13)	0.63
Coronary artery bypass graft (%)	0 (0)	21 (39)	0.02
Valve (%)	8 (80)	26 (48)	0.09
Redo (%)	3 (30)	3 (6)	0.04
Fluid administration			
Crystalloids (ml)	20,000 (1,500 to 3,000)	2,000 (2,000 to 25,000)	0.97
Colloids (ml)	0 (0 to 500)	500 (0 to 500)	0.73
Transfusion (packed red cell units)	2 (0 to 5)	0 (0 to 2)	0.10
Perioperative treatments			
Number of patients treated with ephedrine (%)	4 (40)	23 (42.6)	1.00
Dobutamine total dose (mg)	35 (25 to 60)	0 (0 to 0)	0.001
Furosemid total dose (mg)	5 (0 to 10)	0 (0 to 0)	0.161
AVP and copeptin			
AVP during CPB (pmol/l)	9.3 (1.3 to 11.4)	6.4 (1.8 to 18.4)	0.78
Copeptin during CPB (pmol/l)	50.8 (35.9 to 63.4)	9.4 (3.77 to 36.3)	0.02

Data expressed as the median (25th to 75th percentiles). AVP, arginine-vasopressin; CPB, cardiopulmonary bypass; PCSV, post cardiac surgery vasodilatation.

After surgery, PCSV patients were treated with a moderate dose of norepinephrine (range doses from 0.1 to 1.1 mg/hour). The PCSV patients have a longer extubation time (24 hours (16 to 48) vs. 6 hours (4 to 9.25), *P *< 0.001) and were more frequently treated with dobutamine (range doses from 0 to 10 μg/kg/minute) than other patients (Table 3). The total fluid volume load during surgery and in the first 8 postoperative hours was not significantly different between PCSV patients and other patients (3,250 ml (2,750 to 4,000) vs. 3,000 ml (2,500 to 4,000), respectively; *P *= 0.43). While PCSV patients remained intubated for a longer time than other patients, the propofol cumulative dose over the first 8 hours was not significantly different from other patients (350 mg (380 to 520) vs. 215 mg (155 to 375), respectively; *P *= 0.16). The AVP plasma concentration increase was statistically significant between T0 and H8 and between CPB and H8 in the control group (*P *< 0.001), and between T0 and H8 in the PCSV group (*P *= 0.01), but was significantly lower at H8 in PCSV patients than in controls (Table 3). The plasma copeptin concentration increased significantly between T0 and H8 and between CPB and H8 in both the control and PCSV groups (*P *< 0.001).

**Table 3:** Postoperative data

	PCSV patients (*n *= 10)	Controls (*n *= 54)	*P *value
Hemodynamic treatments			
Dobutamine dose over first 8 hours (μg/kg/minute)	5.8 (5.0 to 6.9)	0 (0 to 0)	0.001
Norepinephrine dose over first 8 hours (mg/hour)	3.5 (1 to 7)	0	0.001
Fluid administration			
Crystalloid dose over 8 hours (ml)	0 (0 to 500)	0 (0 to 0)	0.43
Colloid dose over 8 hours (ml)	500 (250 to 500)	500 (0 to 500)	0.50
Transfusion (packed red cell units)	0 (0 to 2)	0 (0 to 0)	0.32
Biology			
Lactate H0 (mmol/l)	1.8 (1.5 to 2.1)	1.4 (1 to 2)	0.16
Lactate H8 (mmol/l)	2.25 (1.5 to 2.6)	1.45(1.1 to 2.2)	0.06
Osmolarity H8 (mOsmol/l)	288 (284 to 293)	288 (284 to 293)	0.89
Platelets (10^9^/l)	81.5 (63 to 135)	124 (103 to 146)	0.06
Hemoglobin concentration (g/dl)	9.5 (8.8 to 10.6)	10.3 (9.5 to 11.4)	0.16
AVP and copeptin			
AVP H8 (pmol/l)	16.9 (12.9 to 24.5)	42.6 (23.7 to 79)	0.01
Copeptin H8 (pmol/l)	137.5 (118 to 193)	208 (121 to 300)	0.23

Data expressed as the median (25th to 75th percentiles). AVP, arginine vasopressin; H0, arrival in the ICU; H8, eighth postoperative hour; PCSV, post cardiac surgery vasodilatation.

All patients were discharged from the hospital within a few days without serious complications.

**Figure 2:**
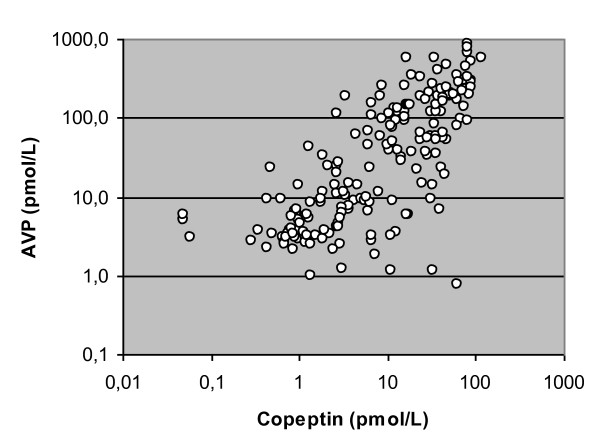
**Correlation analysis between arginine vasopressin and copeptin plasma concentrations**. A Spearman rank-order correlation test has been performed to assess the correlation between arginine vasopressin (AVP) and copeptin plasma concentrations from 191 pairs of measurements. Spearman *r *= 0.76, *P *< 0.001.

The Spearman rank-order correlation test to assess the relation between AVP and copeptin plasma concentrations was performed on the base of 191 pairs. The correlation is significant (*r *= 0.76, *P *< 0.001) (Figure 2).

## Discussion

The present study shows that preoperative copeptin plasma concentration is predictive of PCSV. Increased preoperative copeptin could be a marker of vasopressin system stimulation before surgery, with a relative AVP system deficiency after surgery.

Our results are in agreement with previous publications where low AVP plasma concentrations have been observed in refractory vasodilatory shock when compared with cardiogenic shock shortly after CPB for cardiac surgery [6]. These results extend the likelihood of a vasopressin deficiency to vasodilatation without shock. We did not observe norepinephrine resistance in this small series of patients; however, the so-called catecholamine resistance varies widely between studies and between patients within studies [4,6,7]. As an example, in a series of 40 patients treated with vasopressin for vasodilatory shock the mean administered dose of norepinephrine was 14.4 ± 13.7 μg/minute [6]. The wide standard deviation suggests some patients were considered catecholamine resistant even when a rather small dose of norepinephrine was infused.

The AVP plasma concentration increase during cardiac surgery performed under CPB has been described for years [18,19]. The AVP peak has been related to arterial hypotension during CPB and to postoperative stimulation of AVP release by inflammatory mediators [20,21]. In the present study, AVP and copeptin plasma concentrations increase significantly in both groups. The AVP response to cardiac surgery resembles the postoperative course of other stress hormones, so higher AVP plasma concentrations would have been expected in patients with hypotension when compared with patients with normal blood pressure [6,22]. Although more patients in the norepinephrine-treated group were receiving dobutamine, which may have contributed to the vasodilatation, AVP levels are lower in these patients than expected in cases of significant hypotension (MAP < 60 mmHg) [6]. Similar inadequacy between plasma concentrations of AVP and low blood pressure has been documented in septic shock, where there is some evidence that depletion of endogenous AVP stores follows vigorous stimulation of AVP release from the neurohypophysis [7,8,23].

A chronic stimulation of the vasopressin system that may contribute to AVP store depletion has been demonstrated in chronic heart failure [11-13]. High copeptin plasma levels have been observed in chronic heart failure and acute myocardial infarction; vasopressin production is related to the severity of heart failure [11-13]. Increased plasma levels of copeptin have been linked to excess mortality in chronic heart failure, and this link is observed irrespective of the clinical signs of severity of the disease. In the present study, PCSV patients had lower preoperative left ventricle ejection fraction and hyponatremia, both relevant markers of heart failure. Copeptin plasma concentration is higher in these patients than in controls; copeptin plasma levels are in the range of values already reported in New York Heart Association functional class III and IV patients [11]. Interestingly, the median preoperative copeptin value with a significant prognostic value (9.43 pmol/l) is double the median copeptin value of the normal population, as reported in the literature [12], and also is similar to the control population of the study. The lower response of vasopressin after surgery while the preoperative copeptin concentration was increased may be thus accounted for by a depletion of endogenous AVP stores, as a result of sustained preoperative vasopressin system stimulation.

Although significant, the correlation between AVP and copeptin appears rather weak (*r *= 0.76), probably because direct determination of AVP is liable to generate inconsistencies (short half-life of AVP, 90% of which bound to blood platelets, and temperature sensitivity of the blood sample). These effects have hindered the use of AVP dosage in routine diagnostics. The correlation between AVP and copeptin plasma concentration observed in the study is nevertheless similar to that reported in other studies [5,14]. Generation of copeptin correlates with the release of vasopressin, since they are derived from the same precursor. Copeptin is a stable fragment of the AVP precursor pre-pro-vasopressin and is released together with AVP in a 1:1 stochiometric pattern [24]. The advantages of copeptin are its long stability and that it can be quickly and reliably measured in unprocessed plasma or serum. Copeptin may therefore be used as a surrogate parameter of vasopressin system stimulation [25].

The small number of patients in the present study is a limitation that should be taken into account for interpreting negative results such as the absence of difference in copeptin plasma concentration between PCSV patients and the others after surgery during the AVP peak. The similar copeptin and AVP variations and a marked increase in copeptin after surgery in controls when compared with PCSV patients, however, are in agreement with AVP kinetics, and eventually AVP deficiency in PCVS patients. The plasma AVP measurement is known to be affected by platelet counts [5]. Nevertheless, the significant difference in AVP plasma concentration between the two groups after surgery (twofold higher in untreated patients than in PCSV patients) cannot be accounted for by the slight difference in platelet counts. Moreover, the increase in median copeptin plasma concentration after surgery, which is not influenced by platelet counts, is 10-fold higher in controls than in PCSV patients.

## Conclusions

This preliminary study suggests that patients who experience vasodilatation after cardiac surgery may have a preoperative AVP system stimulation that results in a relative postoperative deficiency. Future trials are needed to confirm the possible relationship between the sustained preoperative AVP stimulation, as assessed by copeptin, and AVP store depletion.

## Key messages

• Copeptin plasma concentration is increased before CPB in patients experiencing PCSV.

• Copeptin is a marker of cardiac failure intensity, which may be useful to identify patients at risk of post cardiac surgery vasoplegia.

• Increased stimulation of the vasopressin system before surgery may contribute to vasopressin deficiency after surgery.

## Abbreviations

AVP: arginine vasopressin; CPB: cardiopulmonary bypass; H8: eighth postoperative hour; MAP: mean arterial pressure; PCSV: post cardiac surgery vasodilatation; T0: before cardiopulmonary bypass.

## Competing interests

JS and NGM are employed by Thermo Fisher Scientific, the company that developed and patented the copeptin assay. The other authors declare that they have no competing interests.

## Authors' contributions

PHC conceived of the study and helped to draft the manuscript. CB carried out the patient data collection and helped to draft the manuscript. JS and NGM performed the copeptin measurements and drafted the manuscript. BA participated in the design of the study and helped to draft the manuscript. GG carried out the immunoassays of vasopressin measurements. All authors read and approved the final manuscript for publication.
